# Assessing the cyclic fatigue resistance and sterilization effects on replica-like endodontic instruments compared to Reciproc Blue

**DOI:** 10.1038/s41598-023-50096-2

**Published:** 2023-12-27

**Authors:** Fernando Antonio Siano dos Reis, Amjad Abu Hasna, Gustavo Ragozzini, Felipe Bernardo de Moura, Tiago Moreira Bastos Campos, Alexandre Sigrist de Martin, Cláudio Antonio Talge Carvalho, Carlos Eduardo Silveira Bueno

**Affiliations:** 1 Faculdade São Leopoldo Mandic, Instituto de Pesquisas São Leopoldo Mandic, Endodontia, Campinas, SP Brazil; 2 Department of Restorative Dentistry, Endodontics Division, Institute of Science and Technology, São Paulo State University (UNESP), Av. Eng. Francisco José Longo Avenue 777, São José dos Campos, SP CEP 12245-000 Brazil; 3 School of Dentistry, Universidad Espíritu Santo, Samborondón, Ecuador; 4 Physics Department, Aeronautics Technological Institute (ITA), São José dos Campos, SP Brazil

**Keywords:** Materials science, Medical research, Preclinical research

## Abstract

This study aimed to evaluate the effect of the number of uses and autoclave sterilization on the cyclic fatigue resistance of four replica-like instruments RC Blue; Only One File Blue; Recip One Blue; and Micro Blue compared to the original system Reciproc Blue (VDW, Munich, Germany). The instruments were analyzed by scanning electron microscope (SEM) before being used in root canal instrumentation (baseline). Fifty molars were instrumented by ten instruments (n=10). After sterilization in an autoclave, the  instruments were analyzed by SEM. This procedure was repeated twice more using different molars, totaling 3 rounds of instrumentation, sterilization and SEM analysis. Then, ten different instruments from each brand were tested for cyclic fatigue resistance. Number of uses data were analyzed using Chi-squared analysis, and cyclic fatigue data were analyzed by one-way Anova followed by Tukey’s test as the data had normal distribution. The fracture times for all systems had no significant difference, but Micro Blue had significantly lower values than the other systems (*p* < 0.05). The SEM analysis showed distortions in the instruments after the 3rd use. Therefore, all tested instruments except of Micro Blue have similar resistance to cyclic fatigue, and all are reliable for use in up to 2-cases.

## Introduction

Endodontic instruments are used with irrigants to clean and shape the root canal system^1^. These instruments, initially, were made of inflexible stainless-steel alloy, however, diverse accidents were reported during the treatment with these instruments^2^. Posteriorly, nickel-titanium (Ni–Ti) alloy was introduced as an alternative material to produce more flexible endodontic instruments. Ni–Ti instruments were improved during the last decades in relation to their design, metallurgical features, mechanical behavior, shaping ability, and kinematics^3,4^. Still, Ni–Ti instruments may fracture because of cyclic fatigue due to excessive use, after numerous cycles of autoclave sterilization^5,6^, or because of torsional failures when it hangs on the dentin wall, while the shank continues rotating^6^.

Kinematic^7,8^ and heat treatment^9–12^, are two technological advancements that led to the populating of Ni–Ti endodontic instruments. The asymmetric oscillatory motion concept, the so-called reciprocating movement, introduced by Yared^7,8^, extends the instrument lifespan by increasing its resistance to fatigue. Besides, the heat treatment that has a significant effect on the flexibility of Ni–Ti endodontic instruments, results in a color change of the endodontic instrument ranging among silver-white, golden-orange, royal-blue, green, dark-gray and brownish-black color surface^11^. Currently, gold, blue, and gray heat-treated alloys are commonly found in commercialized endodontic instruments with different kinematics and mechanical properties^13^.

Reciproc system (VDW, Munich, Germany) was the first launched reciprocating system^14^, according to the manufacturer, it is a one file shaping system of s-shaped cross-section instrument that has a higher resistance to cyclic fatigue and a reduced risk of instrument fracture. Moreover, this system was improved with a heat-treatment technology to be more flexible and more resistant to cyclic fatigue, Reciproc Blue (VDW, Munich, Germany), therefore, offering more secure instrumentation of the root canal system^15^.

Replica-like instruments of different brands were launched in the markets promising similar properties to the original Reciproc Blue system^16^, however, these systems have not been tested. To the best of our knowledge, there are no studies in the literature on the mechanical behavior of replica-like instruments including RC Blue of Dental Perfect (Shenzhen Perfect Medical Instruments, Shenzhen, China), Only One File Blue of Denco (Shenzhen Denco Medical, Shenzhen, China), Recip One Blue of Rogin Dental (Shenzhen Rogin Medical, Shenzhen, China) and Micro Blue of Microdont (Microdont, Shenzhen, China).

Therefore, this study aimed to evaluate the effect of the number of uses and auto-clave sterilization in the cyclic fatigue resistance of Reciproc Blue (VDW, Munich, Germany) and four replica-like endodontic instruments. The null hypothesis of this study was that there is no difference in the mechanical behavior among the evaluated systems.

## Material and methods

### Sample size calculation

Considering the mean and standard deviation of the experimental groups of anterior study^6^, using the site https://www.sealedenvelope.com, in which the significance level α = 5%, the power (1−β) = 80%, standard deviation = 143.7, and equivalence limit = 210, it was found that the sample size required per group = 10.

### Specimens’ selection

A total of one hundred and fifty upper and lower molars, newly extracted due to orthodontic reasons or periodontal disease, were gathered for the purpose of this investigation. The acquisition of these molars was conducted with the approval of the Research Ethics Committee at the Institute of Science and Technology of São Paulo State University (protocol number: 5.940.123). In addition, informed consents were obtained from all subjects and/or their legal guardian. In this study, all methods were performed in accordance with the relevant guidelines and regulations.

Prior to use, the teeth underwent a disinfection process involving 1% sodium hypochlorite (NaOCl) treatment for a duration of 24 h, followed by rinsing with saline solution. Subsequently, the teeth were frozen to preserve them until the commencement of the study.

Inclusion criteria were established as follows: molars that were non-carious, adequately hydrated, possessed a fully formed apex, exhibited no calcification, and had not undergone any prior treatment. Molars not meeting all these specified criteria were excluded from participation in the study.

### Experimental groups

Five different endodontic systems (n = 10) were evaluated in this study:

- Reciproc Blue (#25.08) of VDW (VDW, Munich, Germany);
- RC Blue (#25.08) of Dental Perfect (Shenzhen Perfect Medical Instruments, Shenzhen, China);
- Only One File Blue (#25.08) of Denco (Shenzhen Denco Medical, Shenzhen, China);
- Recip One Blue (#25.08) of Rogin Dental (Shenzhen Rogin Medical, Shenzhen, China); and
- Micro Blue (#25.08) of Microdont (Microdont, Shenzhen, China).

### Scanning electron microscope (SEM)

Two instruments of each brand were examined under a scanning electron microscope (SEM) (Inspect S50, PT Multi Teknindo Infotronika, Jakarta, Indonesia) to assess any fractures or cracks (initial evaluation). Instruments were cleaned with absolute alcohol for 3 min and fixed in a metal stub and examined at magnification (100×, 200×, 500×, 1000×, 2000× and 5000×). After the initial evaluation, the topographic features of the surfaces of these instruments were evaluated after three more times after each instrumentation and autoclave sterilization cycle.

### Auto-clave sterilization effect on number of uses

**Figure 1:**
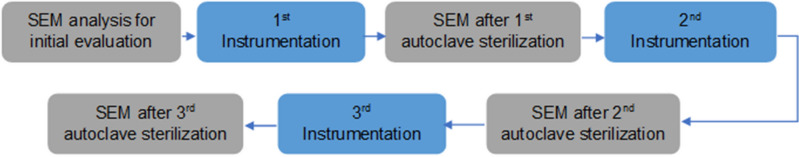
Flowchart of SEM analysis, instrumentation, and autoclave sterilization cycles.

The chosen molars were accessed by a single operator (an endodontist) using the conventional access cavity style. The canals were probed with #10 stainless steel K-files (Dentsply Maillefer, Oklahoma, USA), and the working length was determined to be 1 mm short of the apical foramen. The instrumentation process for each specimen involved ten instruments from each brand (n = 10), connected to the silver VDW endodontic motor (VDW, Munich, Germany) set at the manufacturer's recommended reciprocal motion (RECIPROC ALL). This instrumentation was carried out in three phases, corresponding to the cervical, middle, and apical thirds of the root canal. In terms of irrigation, each third of the canal received 5mL of 2.5% NaOCl, following the manufacturer's instructions, resulting in a total of 15 ml for each canal. This standardized protocol was applied to all root canals. Following this, the instruments underwent thorough cleaning using an ultrasonic cleaner and were then sterilized in an autoclave (Autoclave Vitale Plus 21 Cristofoli Biosafety Equipment, Campo Mourão, PR, Brazil). Subsequently, the instruments underwent another round of SEM analysis. This entire process of instrumentation, autoclave sterilization, and SEM analysis was repeated three times, with each iteration involving 50 teeth, culminating in a total of 150 instrumented molars (Fig. 1).

### Autoclave sterilization

The first step in the sterilization process involved cleaning the endodontic instruments using an ultrasonic cleaner and detergent for a duration of 15 min. Subsequently, they were thoroughly rinsed with water and left to air-dry. Following this, the instruments were carefully placed into autoclave bags or pouches specifically designed for sterilization, and the pouches were sealed according to the manufacturer's instructions.

Once properly packaged, the pouches were arranged in the autoclave chamber with careful attention to avoid overcrowding, ensuring there was adequate spacing between them to facilitate uniform steam penetration and sterilization. The autoclave was then set in accordance with the manufacturer’s guidelines, utilizing steam under a pressure of 30 psi and a temperature range of 121–134 °C for a recommended sterilization duration of 15 min. It was important to configure the autoclave to the appropriate sterilization cycle intended for wrapped instruments. After the autoclave completed its full cycle, encompassing heating, sterilization, and cooling phases, the sterilized instruments were removed from the chamber. To maintain their sterility, they were stored in a clean, dry, and safeguarded environment. This meticulous process ensures the instruments are ready for safe and sanitary use.

### Cyclic fatigue

Different sets of instruments from each brand (n = 10) were coupled to the VDW silver endodontic motor, adjusted to operate with the factory-defined reciprocating motion (RECIPROC ALL).

**Figure 2:**
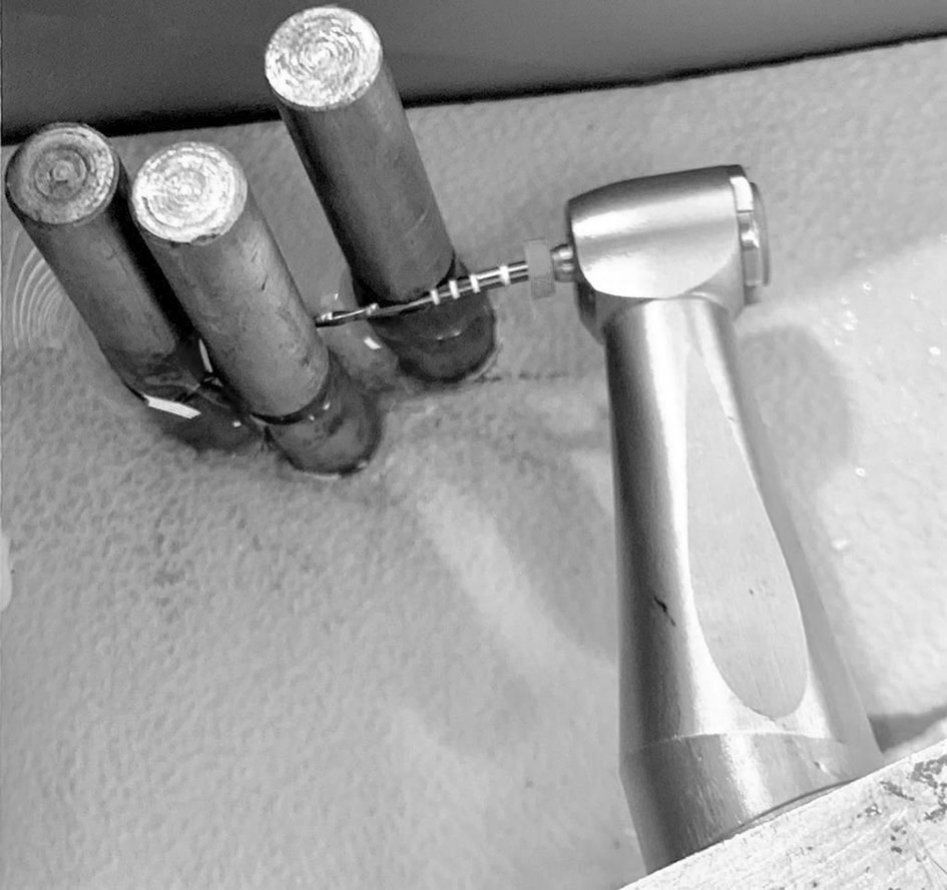
The equipment employed for conducting the cyclic fatigue test.

To replicate the root canal curvature, a plastic base featuring three adjustable stainless-steel pins was utilized. These pins were 6 mm in diameter, 4 cm in length, and had a V-shaped notch measuring 0.5 mm in width. This setup mimicked the canal curvature and was in line with previous studies^6,17^ (Fig. 2). The plastic base was submerged in 200 mL of deionized water within a heating chamber (Kasvi, Taiwan). The water temperature was precisely maintained at 37 ± 0.5 °C, and an infrared thermometer monitored the temperature throughout the testing.

Each instrument underwent testing on a 3 mm bending radius, with a bending angle of 60°. The center of curvature was positioned 4.5 mm from the instrument tip, and the working length for all tested instruments was standardized at 19 mm. The determination of the curvature's radius and angle followed the methodology outlined by Pruett et al.^18^. The motor activated the instruments until separation occurred, with the time of separation being measured in seconds.

### Statistical analysis

Data were analyzed by D'Agostino & Pearson omnibus, Shapiro–Wilk and KS normality tests. The analysis of the number of uses data was conducted using Chi-squared analysis. For the cyclic fatigue data, Anova one-way analysis followed by Tukey's post hoc test was employed as the data presented normal distribution, and there was only one variable to be analyzed. These analyses were executed utilizing GraphPad Prism 5.0 software, with a predetermined significance level of 5%.

## Results

### Auto-clave sterilization effects on number of uses

According to the results reported in Table 1, no statistical difference emerged in the number of fractured instruments after 1st, 2nd and 3rd use among all the tested groups (*P* > 0.05).

**Table 1:** The number of fractures per use.

	Fractures (1st use)	Fractures (2nd use)	Fractures (3rd use)	Total fractures
VDW (Reciproc Blue)	0	0	1	1
Perfect (RC Blue)	0	0	1	1
Denco (Only One File Blue)	0	1	1	2
Rogin (Recip One Blue)	0	0	1	1
Microdont (Micro Blue)	0	0	2	2

### Cyclic fatigue

**Figure 3:**
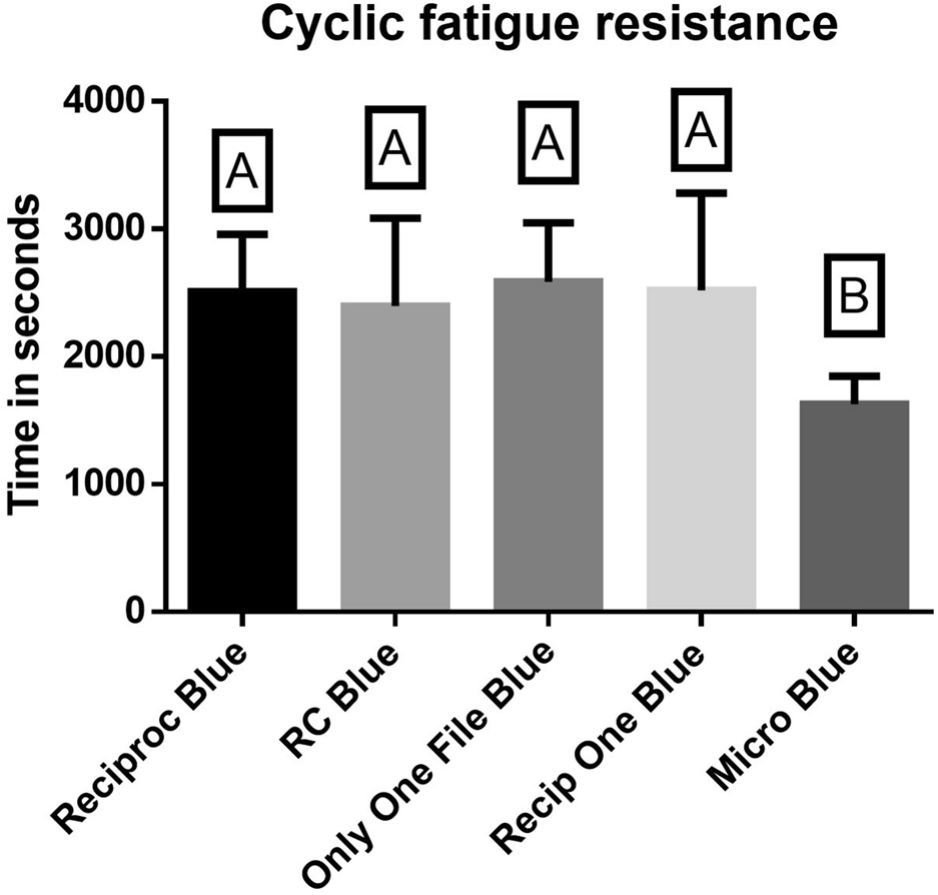
The cyclic fatigue resistance (time to fracture in seconds) of the tested groups. Uppercase letters indicate statistically significant differences.

The outcomes of the cyclic fatigue test are presented in Fig. 3. The fracture times mean values in seconds (s) observed for Reciproc Blue (2509 s), RC Blue (2394 s), Only One File Blue (2584 s), and Recip One Blue (2518 s) exhibited comparable results. However, it's noteworthy that Micro Blue (1626 s) displayed significantly lower values compared to the other systems (*P* < 0.05).

### Scanning electron microscope (SEM)

**Figure 4:**
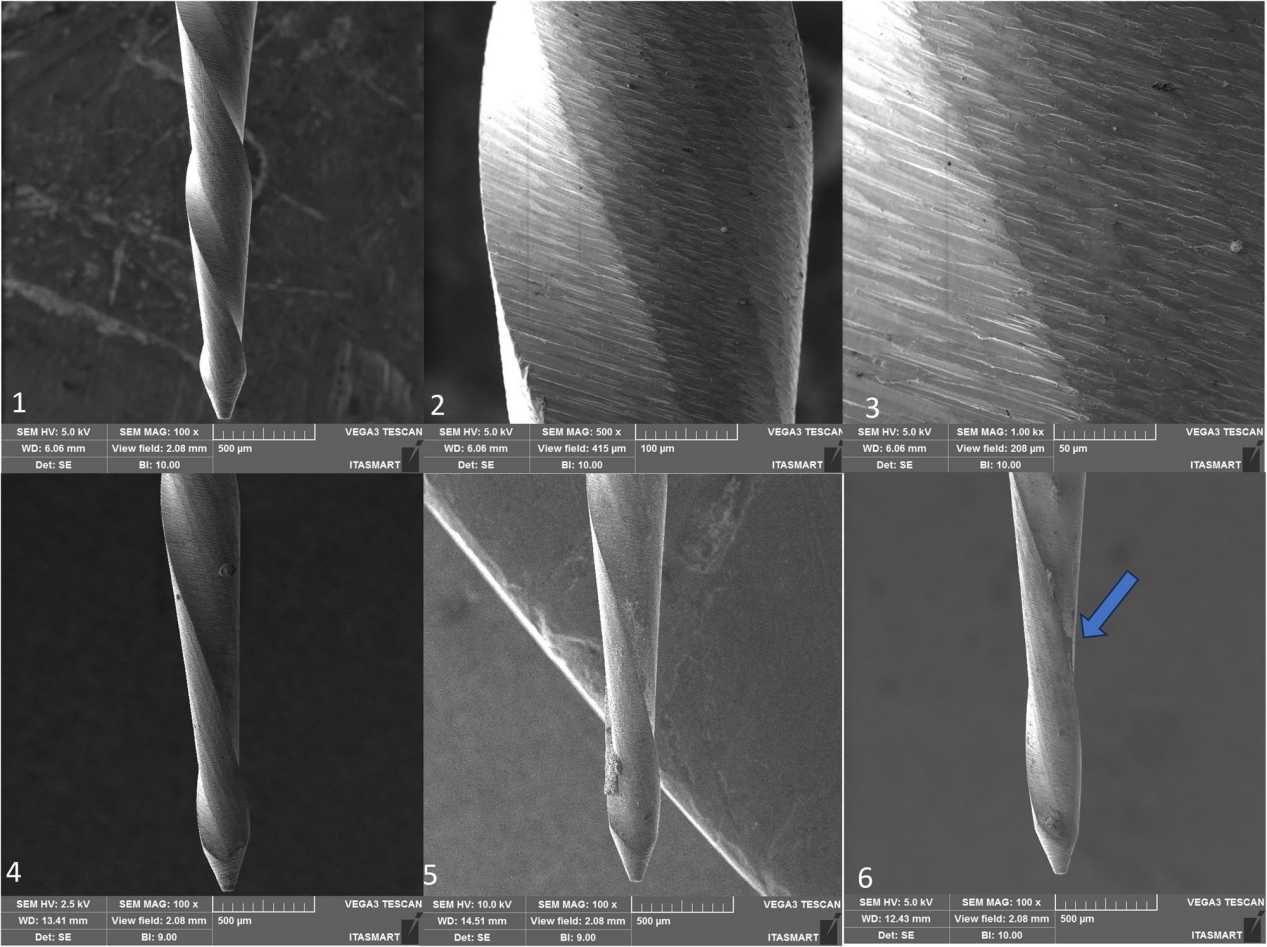
Recirpoc Blue instrument, non-used at 100× (1), 500× (2), 1000× (3), after the first use (4), after the second use (5), and after the third use (6). Legend: Blue arrow indicates distortion.

**Figure 5:**
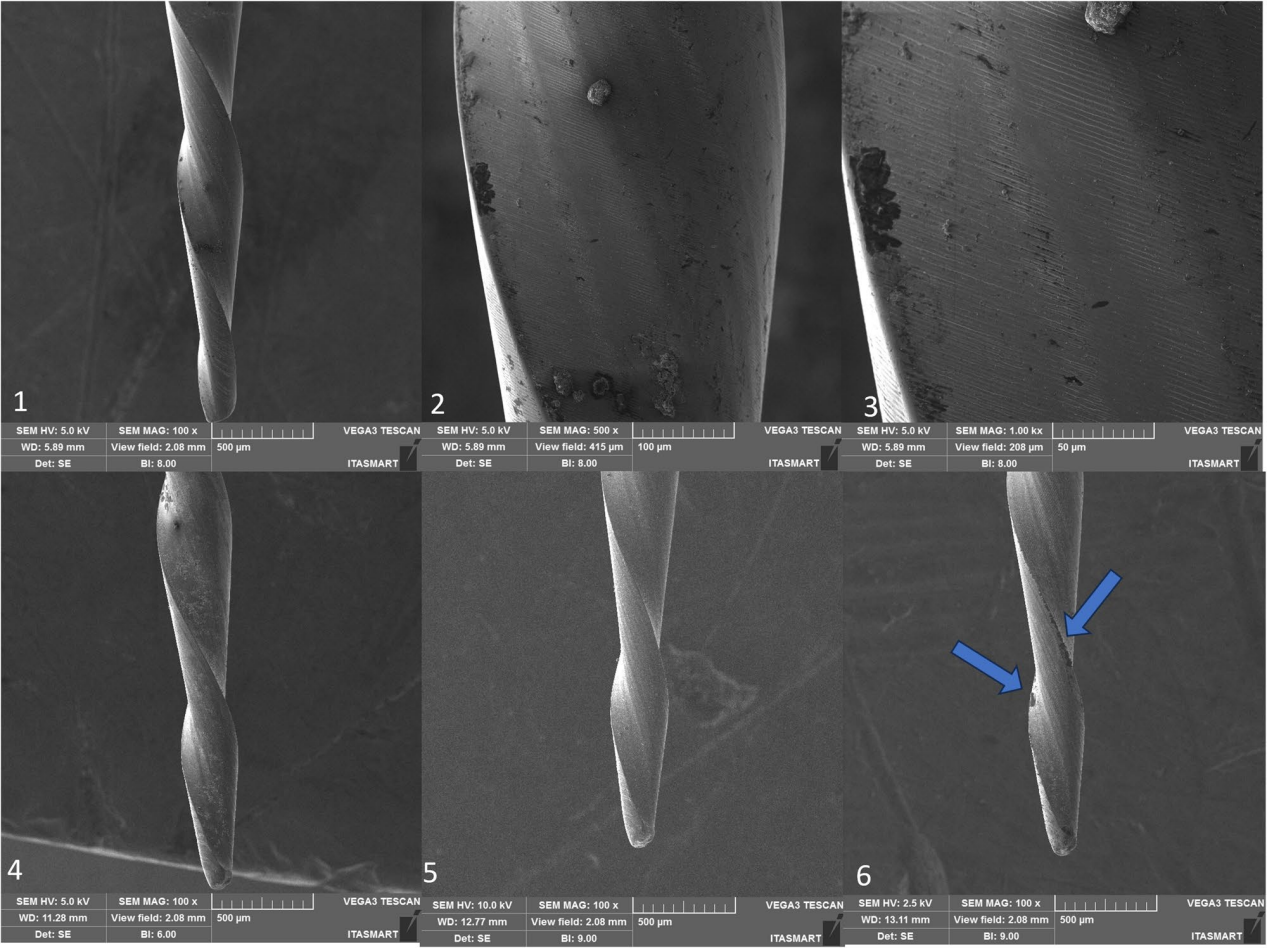
RC Blue instrument, non-used at 100× (1), 500× (2), 1000× (3), after the first use (4), after the second use (5), and after the third use (6). Legend: Blue arrow indicates distortion.

**Figure 6:**
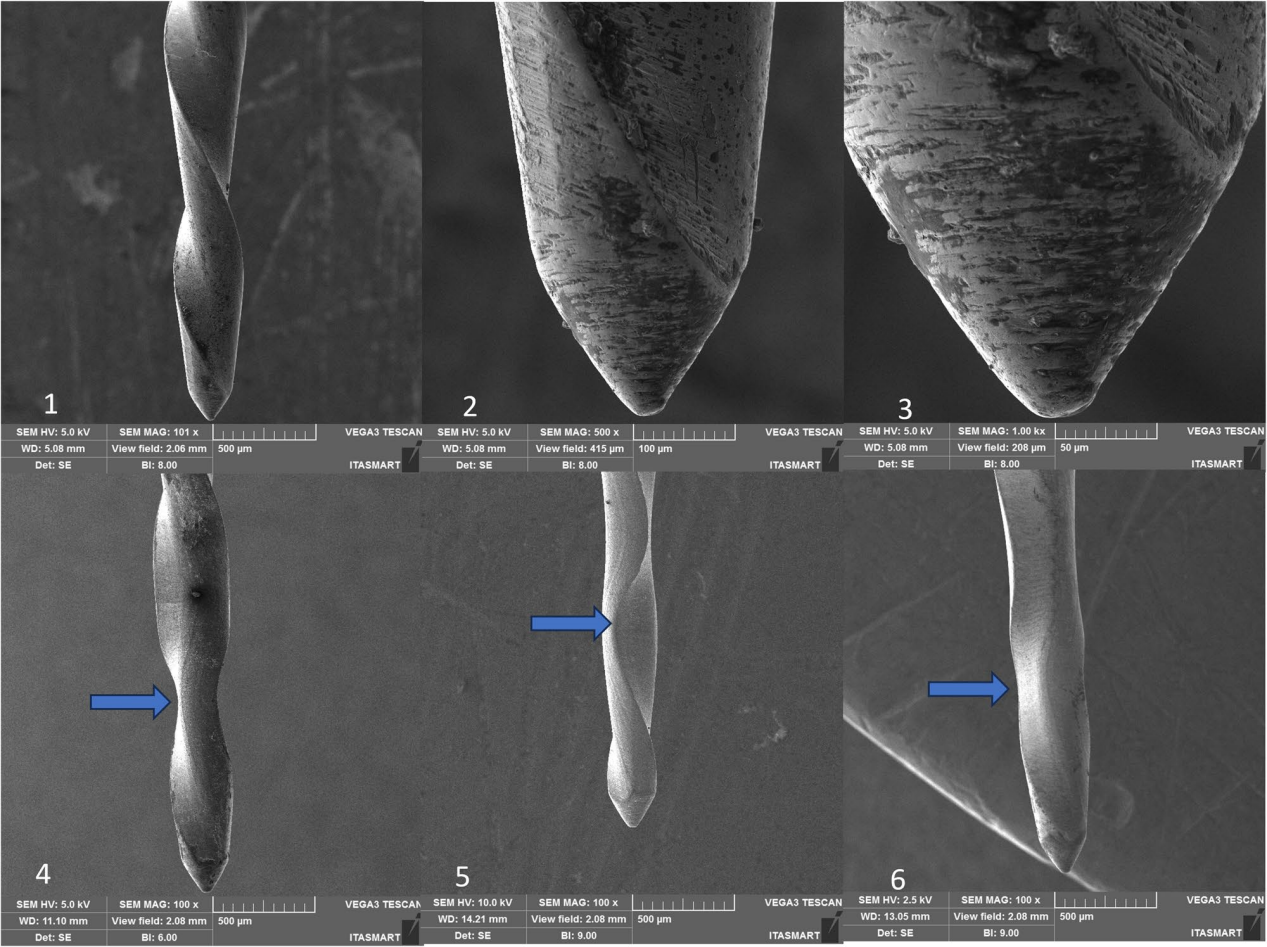
Only One File Blue instrument, non-used at 100× (1), 500× (2), 1000× (3), after the first use (4), after the second use (5), and after the third use (6). Legend: Blue arrow indicates distortion.

**Figure 7:**
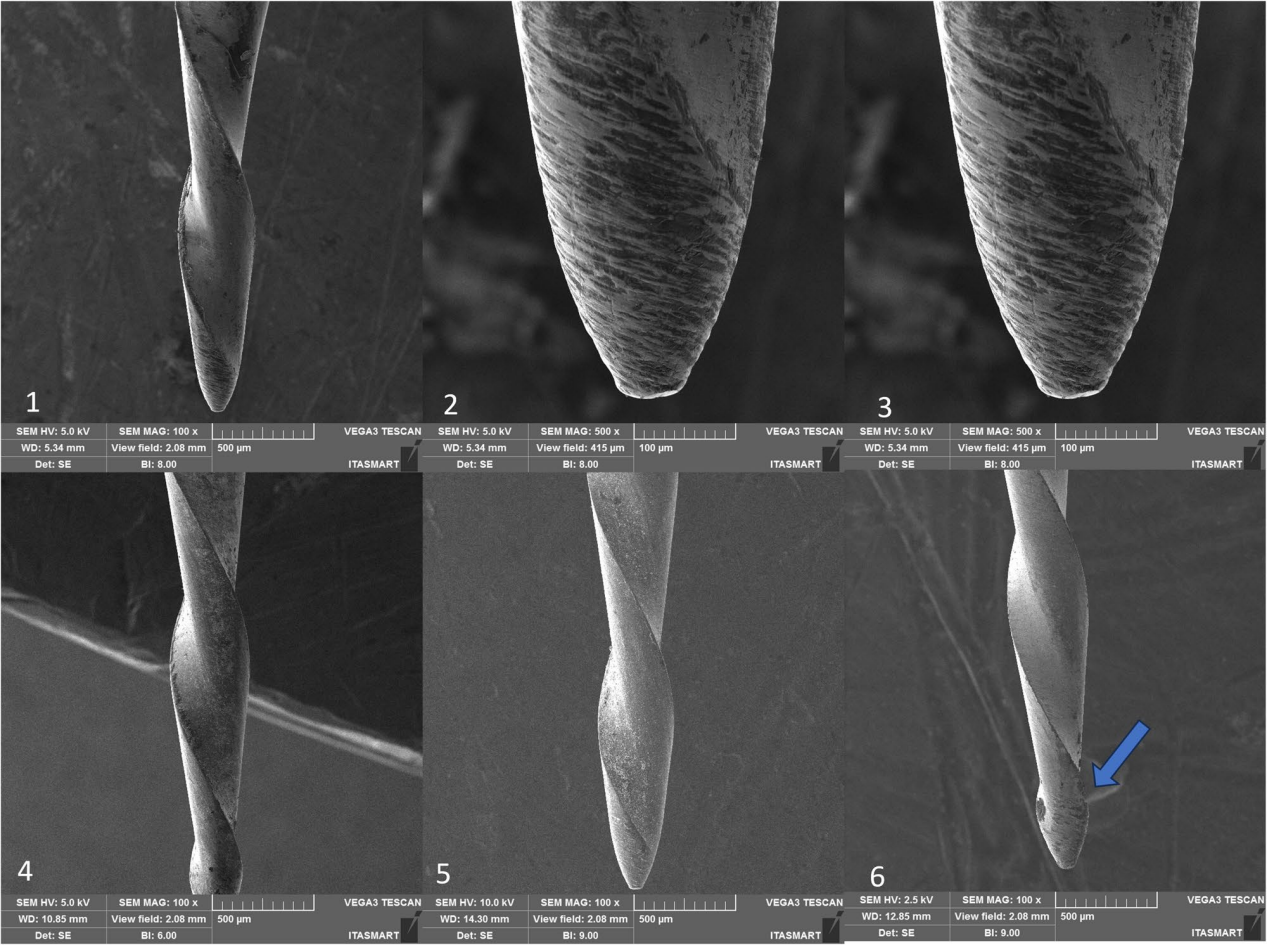
Recip One Blue instrument, non-used at 100× (1), 500× (2), 1000× (3), after the first use (4), after the second use (5), and after the third use (6). Legend: Blue arrow indicates distortion.

**Figure 8:**
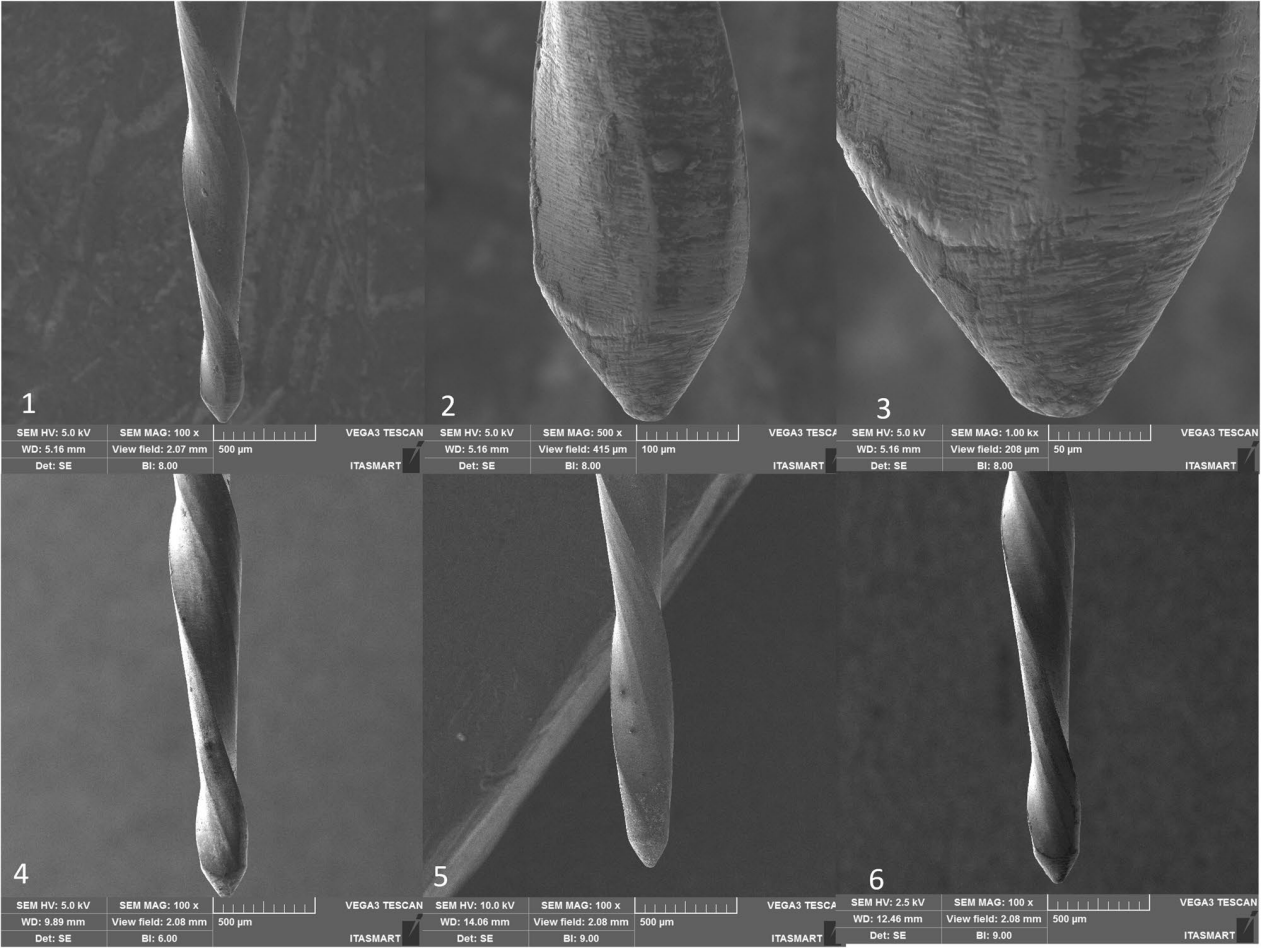
Micro Blue instrument, non-used at 100× (1), 500× (2), 1000× (3), after the first use (4), after the second use (5), and after the third use (6). Legend: Blue arrow indicates distortion.

Within the Reciproc group, the non-utilized instrument displayed satisfactory finishing, yet a deformation became evident after its third use (refer to Fig. 4). Contrastingly, in the RC Blue, Only One File Blue, Recip One Blue, and Micro Blue groups, the finishing quality fell short in comparison to the Reciproc Blue group, in which a small number of defects such as porosities in the cutting edges and flutes could be observed (see Figs. 5, 6, 7, and 8, respectively).

SEM analysis revealed that all instruments subjected to testing displayed uncutting tips. However, the tips were observed to be more rounded in the case of RC Blue and Recip One Blue (Figs. 5 and 7), while Only One File Blue and Micro Blue appeared to have tips that resembled a triangular shape (Figs. 6 and 8).

Additionally, deformations were detected: in the RC Blue group following the third use (Fig. 5), in the Only One File Blue group after the first, second, and third uses (Fig. 6), and in the One Blue group after the third use (Fig. 7). Notably, the Micro Blue group exhibited no instances of distortion (Fig. 8).

## Discussion

A new phenomenon has emerged in the dental products market in the recent years, corporations from emerging economic countries such as China and India began to manufacture and market various types of materials such as mechanized endodontic instruments^19^. According to the manufacturers, all these replica-like instruments are thermically heated Ni–Ti instruments, have two cutting-edge designs (S-shape cross section), and have non-cutting tips. All these instruments should be used in reciprocating kinematics of (RECIPROC ALL), which is the same configuration of the original Reciproc Blue of VDW. None of the manufacturers reported any information about the rotation angle or corrosion of files.

There are some reasons for this, such as lower manufacturing costs, cheap labor, higher production capacity in shorter time and market expansion opportunities. Although some companies have developed innovative instruments for the biomechanical preparation of root canals, others only copy or imitate the physical appearance of original products, the so-called replica-like systems^20^. However, there are still no international regulatory tools that allow the imposition of quality control standards to guide the production of reciprocating or rotating Ni–Ti systems^19^. Therefore, clinicians are often unaware of the risks of using products without a scientific basis. Therefore, this study aimed to provide new information on the mechanical performance of four commercially available replica-like instruments of the original Reciproc Blue system (VDW, Munich, Germany). Cyclic fatigue resistance, autoclave sterilization effect on number of uses, and Scanning Electron Microscope (SEM) of the original Reciproc Blue file were used as controls for the evaluation of the replicalike systems and, considering significant differences emerged in cyclic fatigue test. Thus, the null hypothesis can be partially rejected. Fracture of Ni–Ti instruments during clinical use, due to cyclic fatigue, occurs when the file rotates in a curved canal through repeated cycles of compressive and tensile stresses^21^. The cyclic fatigue parameters represent measures of mechanical resistance, when favorable, they predict a better clinical performance of the instruments, when subjected to this specific stress, reducing the chance of fracture. In the cyclic fatigue test, the Micro Blue system had the shortest fracture time among all instruments, this may be related to its inadequate surface finish^22^, as observed in the SEM analysis.

Cyclic fatigue testing utilizes a static model where the instrument is mounted on a stabilized handpiece and freely rotates in an artificial canal until it fractures, thus transferring these results to the clinician may not be appropriate. However, this test allows avoiding the interference of different variables, it is possible to isolate and analyze the factors individually, increasing the internal validity of the method^23,24^, including the type of dynamic or static movement and the temperature of the test^23,25^. In this study, ambient temperature was chosen for the tests, as this is the temperature at which instruments are normally stored and used in practice. Recently it has been suggested that ambient temperature should be regarded as insignificant and exceed^23^, however, other authors defend the use of body and intracanal temperature^25,26^.

It is possible to observe that most of the instruments submitted to cyclic fatigue tests under body temperature suffer a decrease in their resistance due to the increase in temperature transmitted from the artificial canal to the metallic alloy, which can induce partial or total austenitic formation^20^, but it is unlikely that in the short time that the instrument comes into contact with the dentin, the temperature rises and stabilizes to the point of inducing phase changes in the alloy^27^. Furthermore, the irrigating solution and the thermal insulation of the dentin are also factors that can prevent the instrument from reaching body temperature^20^.

In the present study, it was found that the fracture times for Reciproc Blue, RC Blue, Only One File Blue, and Recip One Blue were similar, but Micro Blue had significantly lower values than the other systems. However, it was not possible to compare the results of the present study with others in the literature as, to the best of our knowledge, this is the first study that compares the cyclic fatigue of these four replica-like systems with the original Recirpoc Blue.

The results regarding the sterilization cycles showed that there was no influence on the estimated number of uses of Reciproc Blue, RC Blue, Only One File Blue, Recip One Blue, and Micro Blue systems. Most fractures were observed after the third sterilization cycle and only one file from the Only One File Blue system fractured after the second cycle. According to the manufacturers, the files are designed to be used only once, they warn of the risks that the reuse of files can cause in the resistance of the instruments and the mechanical effects of reuse have already been demonstrated previously^28^. This study demonstrated that the use of the instruments in up to 2 cases can be considered safe, which agrees with previous studies that indicate that the use of reciprocating files in several cases can be stable and efficient^29,30^.

Still, through SEM we can observe that all the files present irregularities on the surface before sterilization in autoclave, this agrees with previous studies that verified the presence of irregularities and debris on the surface of new endodontic non-used instruments^31,32^ which we attribute to presence of surface roughness from the manufacturing process of NiTi files and their conditioning. Likewise, the instruments must be meticulously checked before use, by operating microscope, to avoid a fracture of the instrument during or preparation of the root canal. After the first use and first autoclaving, the irregularities of the surface of all the groups of the present study reduce. This observation agrees with previous studies^33^, in which it was observed that the surface of Wave One Gold and Reciproc Blue presented less porosity after root canal preparation. This may be related to the wear that occurs during root canal preparation, it is believed that contact areas of instruments subjected to light loads suffer a fine polishing^33^.

Besides, in the present study, SEM analysis revealed that all instruments subjected to testing displayed uncutting tips, these finding confirm the information provided by the manufacturers of these instrument, however, it is important to highlight that the tip design was not unique in all the tested instruments, in which the tips were observed to be more rounded in the case of RC Blue and Recip One Blue (Figs. 5 and 7), these tips design are different of the Reciproc blue tip design which has triangular tip^3^, while Only One File Blue and Micro Blue appeared to have tips that resembled a triangular shape (Figs. 6 and 8).

Until the completion of this work, there were no studies in the literature that had mechanically tested the RC Blue, Only One File Blue, Recip One Blue, and Micro Blue files, therefore more research on the mechanical properties, geometric characteristics, metallic alloy, ability to shaping and cutting efficiency of replicalike instruments must be carried out to understand their safety in use compared to the original systems.

SEM analysis showed that the finishing of the replica-like instruments was not as good as that of the original Reciproc Blue, however, distortions were found in all the tested instruments after the third use, and in Only One File Blue group was found after the first use. Still, it seems that the finishing of the instruments had no effect on their mechanical performance. However, it is essential to note that the presence of microcracks in some of these instruments serves as a warning signal. Such microcracks are indicative of impending instrument fractures, which can result in diminished cutting efficiency and suboptimal performance in terms of operation time and rotation. These microcracks may increase the likelihood of instrument failure during clinical procedures, necessitating careful monitoring and assessment of these dental tools to prevent potential complications. Consequently, it is crucial for clinicians to be vigilant in identifying and addressing instruments displaying microcracks to ensure safe and effective dental procedures^34^.

Lastly, this is a laboratorial study in which the experiments were conducted in controlled laboratory conditions, which might not fully replicate the clinical setting. Real-world variables, such as varying tooth and canal anatomy or clinical techniques, were not considered. Besides, this study suggests that replica-like instruments, when used up to two cases, might be considered safe from a cyclic fatigue perspective. Clinicians may cautiously consider incorporating these instruments into their practice, but further research is needed to confirm their safety in a clinical setting.

Within the limitations of this study, it was found that replica-like instruments were similar to Reciproc Blue in terms of the effect of number of uses and autoclave sterilization on cyclic fatigue resistance. Only the Micro Blue system showed less resistance to cyclic fatigue than the other evaluated systems. The replica-like instruments of the Reciproc Blue files appear to be reliable for use in up to 2 cases. However, more studies are needed to confirm these findings.

## Conclusions

- Reciproc Blue, RC Blue, Only One File Blue and Recip One Blue have similar cyclic fatigue resistance.
- All the tested instruments are reliable for use in up to 2 cases.

## Data Availability

The data used to support the findings of this study are available upon request with the corresponding author d.d.s.amjad@gmail.com.
